# Detection limit of ^89^Zr-labeled T cells for cellular tracking: an in vitro imaging approach using clinical PET/CT and PET/MRI

**DOI:** 10.1186/s13550-020-00667-5

**Published:** 2020-07-14

**Authors:** Laura M. Lechermann, Roido Manavaki, Bala Attili, Doreen Lau, Lorna B. Jarvis, Tim D. Fryer, Nick Bird, Luigi Aloj, Neel Patel, Bristi Basu, Matthew Cleveland, Franklin I. Aigbirhio, Joanne L. Jones, Ferdia A. Gallagher

**Affiliations:** 1 Department of Radiology, University of Cambridge, Cambridge, UK; 2 Cancer Research UK Cambridge Institute, University of Cambridge, Cambridge, UK; 3 Wolfson Brain Imaging Centre, University of Cambridge, Cambridge, UK; 4 Department of Clinical Neurosciences, University of Cambridge, Cambridge, UK; 5 Department of Nuclear Medicine, Addenbrooke’s Hospital, Cambridge, UK; 6 GSK Medicines Research Centre, Gunnels Wood Road, Stevenage, UK; 7 Department of Oncology, University of Cambridge, Cambridge, UK

**Keywords:** PET, Cell labeling, Cell tracking, Zirconium-89, Detection limit

## Abstract

**Purpose:**

Tracking cells in vivo using imaging can provide non-invasive information to understand the pharmacology, efficacy, and safety of novel cell therapies. Zirconium-89 (*t*_1/2_ = 78.4 h) has recently been used to synthesize [^89^Zr]Zr(oxinate)_4_ for cell tracking using positron emission tomography (PET). This work presents an in vitro approach to estimate the detection limit for in vivo PET imaging of Jurkat T cells directly labeled with [^89^Zr]Zr(oxinate)_4_ utilizing clinical PET/CT and PET/MRI.

**Methods:**

Jurkat T cells were labeled with varying concentrations of [^89^Zr]Zr(oxinate)_4_ to generate different cell-specific activities (0.43–31.91 kBq/10^6^ cells). Different concentrations of labeled cell suspensions (10^4^, 10^5^, and 10^6^ cells) were seeded on 6-well plates and into a 3 × 3 cubic-well plate with 1 cm^3^ cubic wells as a gel matrix. Plates were imaged on clinical PET/CT and PET/MRI scanners for 30 min. The total activity in each well was determined by drawing volumes of interest over each well on PET images. The total cell-associated activity was measured using a well counter and correlated with imaging data. Simulations for non-specific signal were performed to model the effect of non-specific radioactivity on detection.

**Results:**

Using this in vitro model, the lowest cell number that could be visualized on 6-well plate images was 6.8 × 10^4^, when the specific activity was 27.8 kBq/10^6^ cells. For the 3 × 3 cubic-well, a plate of 3.3 × 10^4^ cells could be detected on images with a specific activity of 15.4 kBq/10^6^ cells.

**Conclusion:**

The results show the feasibility of detecting [^89^Zr]Zr(oxinate)_4_-labeled Jurkat T cells on clinical PET systems. The results provide a best-case scenario, as in vivo detection using PET/CT or PET/MRI will be affected by cell number, specific activity per cell, the density of cells within the target volume, and non-specific signal. This work has important implications for cell labeling studies in patients, particularly when using radiosensitive cells (e.g., T cells), which require detection of low cell numbers while minimizing radiation dose per cell.

## Introduction

Cell therapy is emerging as a living drug for the treatment of a range of conditions such as genetic diseases, cancer, and autoimmune disorders. It has the potential to radically change medical management and is the subject of considerable ongoing preclinical and translational development. Kymriah™ and Yeskarta™, two cell-based gene therapies for the treatment of acute lymphoblastic and diffuse large B cell lymphoma (DLBCL), are two promising examples of cellular therapies. Despite their promise, very few cell products have been approved for clinical use to date [1].

In order to successfully implement cell therapies and translate them into clinical practice, a better understanding of their biodistribution and persistence after administration is required. The success of cellular therapy depends on the precise dosing, timing, and the spatial distribution of administered cells to the site of action in the body. Non-invasive, in vivo real-time tracking of cells via imaging can provide much-needed information and help optimize cell therapies both for discovery research and clinical development.

Therapeutic cells can be labeled both directly (i.e., in vitro) or indirectly (i.e., in vivo) and be imaged non-invasively using a range of techniques including single-photon emission computed tomography (SPECT), positron emission tomography (PET), magnetic resonance imaging (MRI), and optical imaging [2]. Direct labeling of leukocytes using technetium-99m or indium-111 for SPECT imaging has been a routine clinical procedure to image infection and inflammation since the 1980s. ^111^In-SPECT has also been used for therapeutic cell tracking in cancer patients [3–6], while indirect labeling approaches have been employed for in vivo cellular PET imaging. For example, 9-[4-[^18^F]fluoro-3-(hydroxymethyl)butyl]guanine ([^18^F]FHBG) has been used to track genetically modified therapeutic cells in human studies [7, 8]. Compared to other imaging modalities, PET has the potential to provide superior sensitivity for cell tracking [9, 10].

Zirconium-89 (*t*_1/2_ = 78.4 h) has emerged recently as a promising PET radioisotope for direct cell labeling [11–13]. With its long half-life and residualizing properties in cells, zirconium-89 lends itself to longitudinal cell imaging applications which require extended in vivo circulation times and detection over several days [14]. [^89^Zr]Zr(oxinate)_4_ is a lipophilic compound that can enter the cell by passive transport across the cell membrane, exploiting a similar mechanism to that used to label autologous leukocytes with indium-111 for clinical cell imaging [15, 16]. [^89^Zr]Zr(oxinate)_4_-PET holds promise to improve upon the sensitivity limitations encountered with indium-111 SPECT [9, 17], and clinical imaging trials of this approach are currently on-going.

A key issue for cell tracking with these imaging methods is sensitivity, i.e., the need to achieve sufficient radioactivity density of labeled cells in the target region for detection. This is a particular problem for radiosensitive cells, such as T cells, whose survival is compromised by high-density radiolabeling. Estimating the radioactivity density of labeled cells required for detection will be useful in determining the radiolabeling density that provides the best compromise between cell survival and in vivo detection.

To date, very few studies have investigated the limits of detecting cells using PET [18–20]. This work is the first aimed to address the sensitivity issue for cell tracking of radiosensitive T cells directly labeled with very low amounts of [^89^Zr]Zr(oxinate)_4_. An in vitro approach was employed, with cell viability assessed alongside detection on clinical PET systems as a function of cell number and radioactivity exposure per cell. To our knowledge, this is the first study to examine the detection limit of ^89^Zr-labelled cells on human PET systems.

## Materials and methods

All chemicals, unless otherwise noted, were acquired from Sigma-Aldrich and used without further purification. Human serum was purchased frozen from Sigma-Aldrich. All water used was ultrapure (> 18.2 MΩcm^−1^). Zirconium-89 was supplied as Zr^4+^ in a 1-M oxalic acid (PerkinElmer) and was produced at the BV cyclotron Amsterdam via the ^89^Y(p,n)^89^Zr reaction. Activity measurements were made using a CRC-55t dose calibrator (Capintec Inc. Florham Park, NJ) with a calibration factor of 493 for Zirconium-89.

### Cell culture

Jurkat cells, a human lymphoblast cell line (ATCC, TIB-152TM, LGC), were cultured in Roswell Park Memorial Institute medium (RPMI, Gibco), supplemented with 10% (v/v) fetal bovine serum (Thermo Fisher), 200 U/l penicillin, 0.1 g/l streptomycin, 10 mM HEPES, and 2 mM L-glutamate. Jurkat cells were maintained in suspension in a standard CO_2_ incubator (5% CO_2_ v/v).

### [^89^Zr]Zr(oxinate)_4_ synthesis

The [^89^Zr]Zr(oxinate)_4_ complex was synthesized in an aqueous solution according to a published literature procedure [21] (Supporting Information Section 1). The radiochemical purity and yield of [^89^Zr]Zr(oxinate)_4_ was monitored using silica gel impregnated instant thin-layer chromatography paper (ITLC-SG; Agilent Technologies) in ethyl acetate (Supporting Fig. S1 and S2).

### [^89^Zr]Zr(oxinate)_4_ labeling of Jurkat T cells and determination of viability

**Fig. 1:**
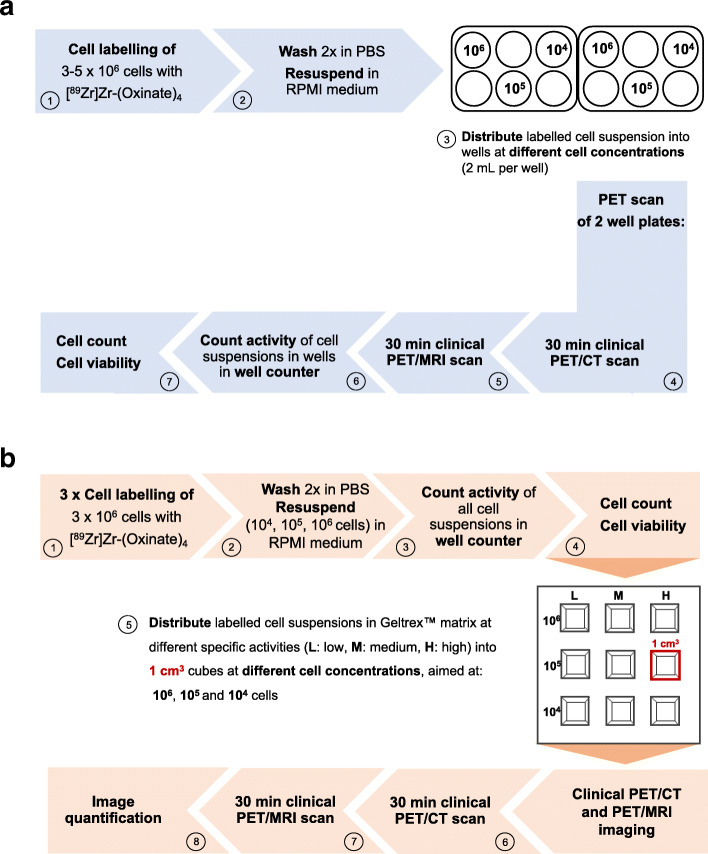
Schematic overview of the setup for the **a** 6-well plate experiments and **b** 3 x 3 cubic-well plate experiment

3–5 × 10^6^ Jurkat cells were washed with phosphate-buffered saline (PBS) and re-suspended in 1.5 mL PBS or complete RPMI medium at room temperature. One hundred fifty-four to 430 kBq of [^89^Zr]Zr(oxinate)_4_ solution was incubated with the respective cell solutions for 30 min at room temperature in a scintillation vial. After incubation, cells were centrifuged and washed twice with PBS. The pellet and corresponding supernatants were measured in a Triathler well counter (HIDEX, Turku, Finland) to calculate the uptake efficacy [%]. Cells were suspended in complete RPMI medium subsequently for further experiments. Cell viability was determined by trypan blue exclusion. Unlabeled Jurkat cells were used as a control to examine changes in cell viability before and after each 6-well plate scan or post-labeling for the 3 × 3 cubic-well plate. An overview of the detailed experiment setup is shown in Fig. 1.

### Detection limit experiment

To explore the detection limit of ^89^Zr-labeled cells on a human PET scanner, ^89^Zr-labeled Jurkat cells were seeded in RPMI medium into 6-well plates at different specific activities, ranging from 10^4^ to 10^6^ cells per well. Additionally, varying concentrations of ^89^Zr-labeled cells suspended in Geltrex™ matrix were placed into a 3 × 3-well plate containing 1 cm^3^ cubic wells in order to examine the effect of cell density on detection. The 6-well plates and 3 × 3 cubic-well plate were imaged on clinical PET/CT (GE Discovery 690) and PET/MR (GE SIGNA) systems (GE Healthcare, Waukesha, USA). The total radioactivity (kBq) in each of the cell suspensions was determined by well counter measurements. The specific activity (SA), defined here as the radioactivity per 10^6^ cells (kBq/10^6^ cells), was calculated for each well based on the corresponding well counter measurement divided by the total number of cells as counted in a hemocytometer. For the 1-cm^3^ cubic wells, cell density (10^6^ cells/mL) was defined as the number of cells contained in the 1-mL well volume. A detailed account of the experimental procedure is provided in the Supporting Information Section 4.

### ^89^Zr-labeled cell PET imaging

PET/CT scanning of the well plates was performed for 30 min directly followed by a 30-min acquisition on PET/MRI. Detailed acquisition parameters are provided in Supporting Information (Sections 5 and 6; Fig. S4).

### PET image analysis

For each experiment, the PET image from the PET/MRI scan was rigidly registered to its PET/CT counterpart, and both were resampled to the space of the CT image (1 × 1 × 1 mm^3^ voxels). For each 6-well plate, utilizing the CT as guidance, cylindrical volumes of interest (VOIs) were defined for each well containing ^89^Zr-labeled cells by manually placing circular regions of 3.4-cm diameter on all contiguous coronal slices encompassing the activity distribution on PET images. Corresponding background VOIs were generated by placing similarly sized cylindrical VOIs within the three empty wells in each 6-well plate. In a similar manner, cubic VOIs (1 cm^3^) were defined for each well of the 3 × 3 cubic-well plate, with three identically sized background VOIs defined in the area between the 3 × 3 cubic-well plate and the outer plastic case (Supporting Fig. S4**)**. The total activity (kBq) in each well was calculated by summing the radioactivity concentration (kBq/mL) within the defined VOI and multiplying by the VOI volume (mL).

As a measure of detectability, the contrast-to-noise ratio (CNR) for wells containing ^89^Zr-labeled cells was calculated according to Eq. 1:

1 $$ {\mathrm{CNR}}_{\mathrm{well}}=\frac{\mu_{\mathrm{well}}-{\mu}_{\mathrm{background}}}{\sigma_{\mathrm{background}}} $$

where *μ*_well_ and *μ*_background_ refer to the mean radioactivity concentrations in each ^89^Zr-containing well VOI and for the background VOIs respectively, and *σ*_background_ is the standard deviation of voxel values in the background VOIs. Image analysis was performed in Analyze 12.0 (AnalyzeDirect Inc., Overland Park, USA).

Calculation of the lower limit of detection (LLD), defined as the minimum number of cells required for detection on imaging, was performed by regressing specific activity vs. CNR for all wells across all experiments to obtain the 95% upper confidence interval (CI) limit on CNR. The upper 95% CI value for CNR was subsequently substituted into the linear regression equation of CNR vs. cell number per well to calculate LLD.

The detection probability of ^89^Zr-labeled cell suspensions on PET/CT and PET/MRI as a function of cell number was estimated through binomial logistic regression using Eq. 2:

2 $$ \mathrm{Detection}\ \mathrm{probability}=\frac{1}{1+{e}^{-\left({\beta}_0+{\beta}_1x\right)}\ } $$

where *β*_*0*_ and *β*_*1*_ refer to the logistic regression coefficients and *x* denotes the cell number [18].

Prior to logistic regression, binary classification of PET/CT and PET/MR image CNR was performed using the Rose criterion (i.e., classification using a threshold of CNR = 5). To investigate the effect of surrounding background radioactivity on the detection probability of ^89^Zr-labeled cells, eight different background levels corresponding to 10–80% of the radioactivity concentrations in each ^89^Zr-containing well were simulated, with *σ*_background_ estimated assuming a Poisson noise model. Simulations were performed in Matlab 2016b (Mathworks Inc., Natick, NA, USA) and resulted in 248 synthetic datasets.

For each 1 cm^3^ well of the 3 × 3 cubic-well plate, a recovery coefficient (RC) was calculated according to Eq. 3:

3 $$ \mathrm{Recovery}\ \mathrm{coefficient}=\frac{{\left(\mathrm{Measured}\ \mathrm{activity}\right)}_{\mathrm{well}}-{\left(\mathrm{Measured}\ \mathrm{activity}\right)}_{\mathrm{background}}}{{\left(\mathrm{Known}\ \mathrm{activity}\right)}_{\mathrm{well}}-{\left(\mathrm{Known}\ \mathrm{activity}\right)}_{\mathrm{background}}} $$

where Measured activity corresponds to the total activity in the well or background VOIs as measured on the PET images and Known activity refers to the total activity of the cell suspensions as measured in the well counter with (Known activity)_background_ = 0 kBq.

To investigate the effect of cell density and specific activity on the recovery coefficient, the following model was fitted to the 3 × 3 cubic-well plate data:

4 $$ {RC}_{ij}={a}_i\log \left({CD}_{ij}\bullet {SA}_{ij}\right)+{\left({\gamma}_0\right)}_{ij}+{\epsilon}_{ij} $$

where CD and SA refer to the cell density and specific activity of well *j* = 1,...,9 when using scanner *i* = 1 (PET/CT) or *i* = 2 (PET/MRI); *α*_*i*_ and *γ*_0_ are the slope and intercept respectively; and *ε*_i*j*_ are within-group (i.e., scanner) errors. Fitting was performed using a non-linear mixed effects model implemented in Matlab 2016b, with fixed effects for the product of cell density and specific activity, and random effects accounting for differences in RC between the two scanners.

### Statistical analysis

Statistical analysis was performed using GraphPad Prism 8.0; (GraphPad Software Inc., La Jolla, USA). Results are presented as mean ± standard deviation (SD) or median [range] as appropriate. The Anderson-Darling test was used to assess distribution normality. Means between the two groups were compared using Student’s two-tailed *t* test, whereas the Kruskal-Wallis *H* test was used for comparison when more than two groups were compared. Correlations between continuous variables were assessed using the Pearson correlation coefficient (*r*). Bland-Altman analysis was used to evaluate differences in well activity and CNR values between PET/CT and PET/MRI. *p* values < 0.05 were considered statistically significant.

## Results

Eleven cell labeling experiments were performed with different Zirconium-89 batches for [^89^Zr]Zr(oxinate)_4_ tracer synthesis, using Jurkat cells with the same passage number for each experiment. Overall, 11 and 12 6-well plates with 33 and 36 wells containing ^89^Zr-labeled cells were scanned within 6 independent experiments using PET/CT and PET/MRI, respectively. Results from five wells were excluded for both PET/CT and PET/MRI owing to pipetting errors (three wells) and inability to quantify the cell number (two wells). The 3 × 3 cubic-well plate containing ^89^Zr-labeled cells in Geltrex™ matrix was scanned once each on PET/CT and PET/MRI.

### [^89^Zr]Zr(oxinate)_4_ synthesis and labeling of Jurkat T cells

The [^89^Zr]Zr(oxinate)_4_ complex was synthesized in an aqueous solution at a mean radiochemical yield of 93.5% ± 3.1 (SD, *n* = 8) as indicated by thin-layer chromatography and was used for cell labeling without further purification. The labeling efficiency measured after a 30-min incubation period ranged from 5.1 to 33.3% of the added activity. Labeling and imaging did not significantly affect the cell viability throughout the experiment (Supporting Fig. S5).

### Imaging and detection limit of ^89^Zr-labeled cells

**Fig. 2:**
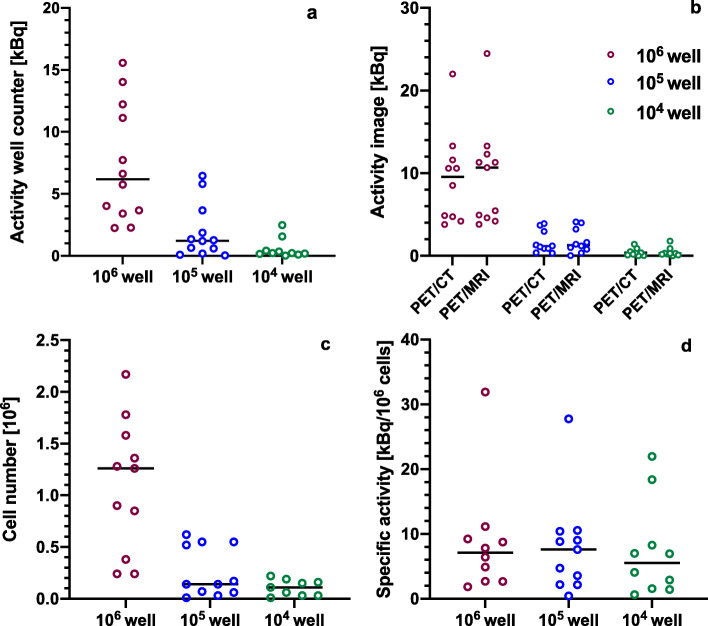
Individual value plots and median of **a** total activity [kBq] in 10^6^, 10^5^, and 10^4^ wells as measured by the well counter; **b** total activity [kBq] in 10^6^, 10^5^, and 10^4^ wells as obtained from PET images acquired using PET/CT and PET/MRI; **c** cell number in 10^6^, 10^5^, and 10^4^ wells; and **d** specific activity [kBq/10^6^ cells] in different wells

For the 6-well plates, the total activity per well ranged from 0.02 to 15.57 kBq and 0.01 to 24.47 kBq as measured by the gamma counter and on PET imaging, respectively (Fig. 2a, b). The median cell number in the 10^6^, 10^5^, and 10^4^ wells was 1.08 × 10^6^, 1.45 × 10^5^, and 8.50 × 10^4^ respectively (Fig. 2c) with specific activities ranging between 0.4 and 31.91 kBq/10^6^ cells (Fig. 2d). No statistically significant difference was observed between the median specific activities in different wells (*p* = 0.71). For the 3 × 3 cubic-well plate, the total activity per 1 cm^3^ well ranged from 0.01 to 12.22 kBq on PET imaging and 0.00 to 9.95 kBq as measured by the gamma counter. Specific activities ranged between 0.14 and 30.63 kBq/10^6^ cells. Detailed information can be found in Supporting Tables S2 and S3.

**Fig. 3:**
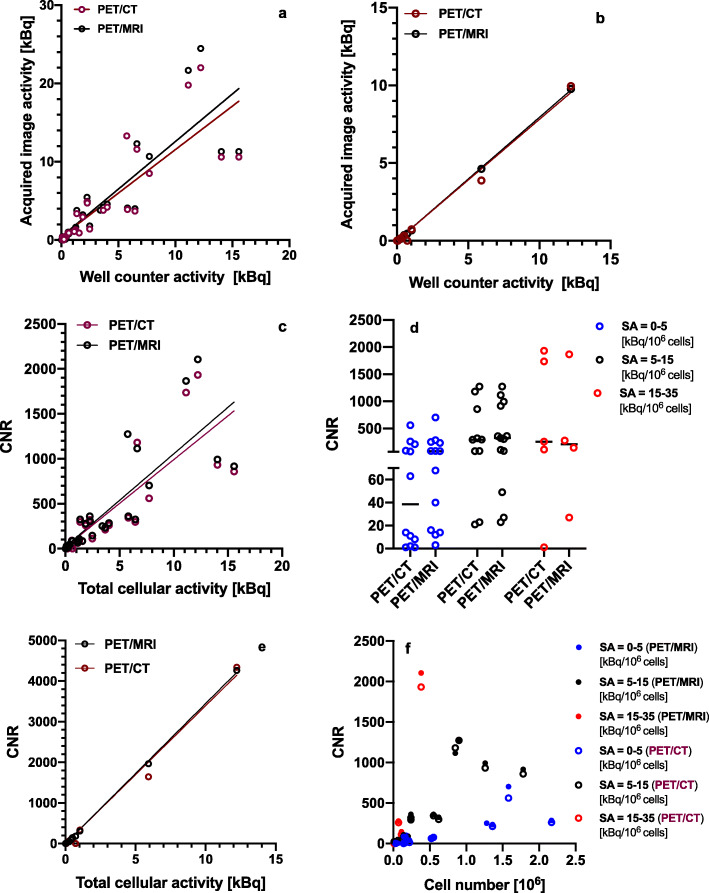
Pearson correlation between the total cell-associated activity [kBq] measured in the well counter and on PET images for **a** 6-well plates and **b** 3 × 3 cubic-well plate. **c** Pearson correlation of PET/CT and PET/MRI CNR vs. total cellular activity [kBq] for 6-well plates. **d** Individual value plot and median of CNR per specific activity category for both PET/CT and PET/MRI for 6-well plates. **e** Pearson correlation of PET/CT and PET/MRI CNR vs. total cellular activity [kBq] for 3 × 3 cubic-well plate. **f** Scatter plot of PET/CT and PET/MRI well CNR vs. cell number per specific activity category. CNR: contrast-to-noise ratio

The total activity in each well as determined from PET/CT and PET/MRI images showed a strong correlation with the total cell-associated activity as measured by the well counter (6-well plates: PET/CT: *r* = 0.84, *p* < 0.0001; PET/MRI: *r* = 0.85, *p* < 0.0001; 3 × 3 cubic-well plate: PET/CT: *r* = 0.995, *p* < 0.0001; PET/MRI: *r* = 0.999, *p* < 0.0001, Fig. 3a, b). Well CNRs ranged from 0 to 2106 for the 6-well plates and 0 to 4341 for the 3 × 3 cubic-well plate. A significant positive correlation was observed between CNR and the total cell-associated activity (6-well plates: PET/CT: *r* = 0.82, *p* < 0.0001; PET/MRI: *r* = 0.83, *p* < 0.0001; Fig. 3c, d; 3 × 3 cubic-well plate: PET/CT: *r* = 0.994, *p* < 0.0001; PET/MRI: *r* = 0.999, *p* < 0.0001, Fig. 3e). Similarly, well CNRs correlated positively with specific activity (6-well plates: PET/CT: *r* = 0.44, *p* = 0.02; PET/MRI: *r* = 0.49; *p* = 0.006, Supporting Fig. S6a; 3 × 3 cubic-well plate: PET/CT: *r* = 0.85, *p* = 0.004; PET/MRI: *r* = 0.82; *p* = 0.007, Supporting Fig. S6b). Furthermore, a significant positive correlation between the CNR and cell number for each well could be observed within each specific activity group for both scanners (Fig. 3f). For PET/CT, correlation coefficients for each specific category group were as follows: 0–5 kBq/10^6^ cells: *r* = 0.81 (*p* = 0.003); 5–15 kBq/10^6^ cells: *r* = 0.80 (*p* = 0.001); 15–35 kBq/10^6^ cells: *r* = 0.98 (*p* = 0.02), whereas for PET/MRI, these were as follows: 0–5 kBq/10^6^ cells: *r* = 0.79, *p* = 0.0008; 5–15 kBq/10^6^ cells: *r* = 0.83, *p* = 0.0005; 15–35 kBq/10^6^ cells: *r* = 0.98, *p* = 0.02.

PET/CT and PET/MRI results strongly correlated for both total well activity and CNRs (Supporting Fig. S8a and S8c). Bland-Altman analysis indicated good agreement between PET/CT and PET/MRI indices of total well activity and CNRs (Supporting Fig. S8b and S8d).

**Fig. 4:**
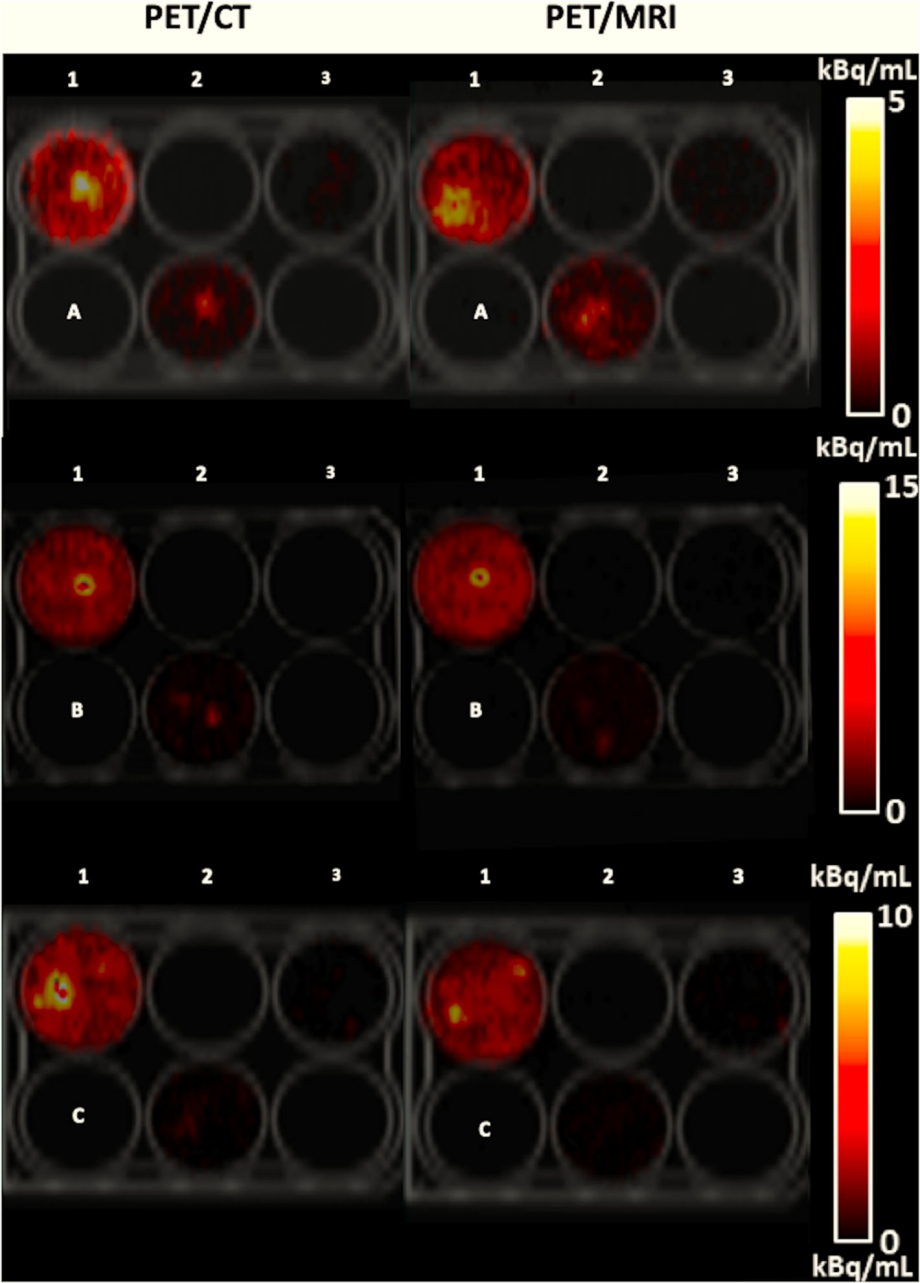
PET images of 6-well plates containing ^89^Zr-labeled Jurkat cells acquired using PET/CT and PET/MRI together with their corresponding co-registered CT image. Cell numbers and specific activities per well (kBq/10^6^ cells; indicated parenthetically) were as follows: (A1) 1.36 × 10^6^ cells (2.70). (A2) 5.20 × 10^5^ cells (2.18). (A3) 1.45 × 10^5^ cells (1.57). (B1) 3.83 × 10^5^ cells (31.91). (B2) 6.75 × 10^4^ cells (27.77). (B3) 1.00 × 10^4^ cells (18.37). (C1) 8.45 × 10^5^ cells (7.83). (C2) 1.65 × 10^5^ cells (3.57). (C3) 6.10 × 10^4^ cells (6.93)

**Fig. 5:**
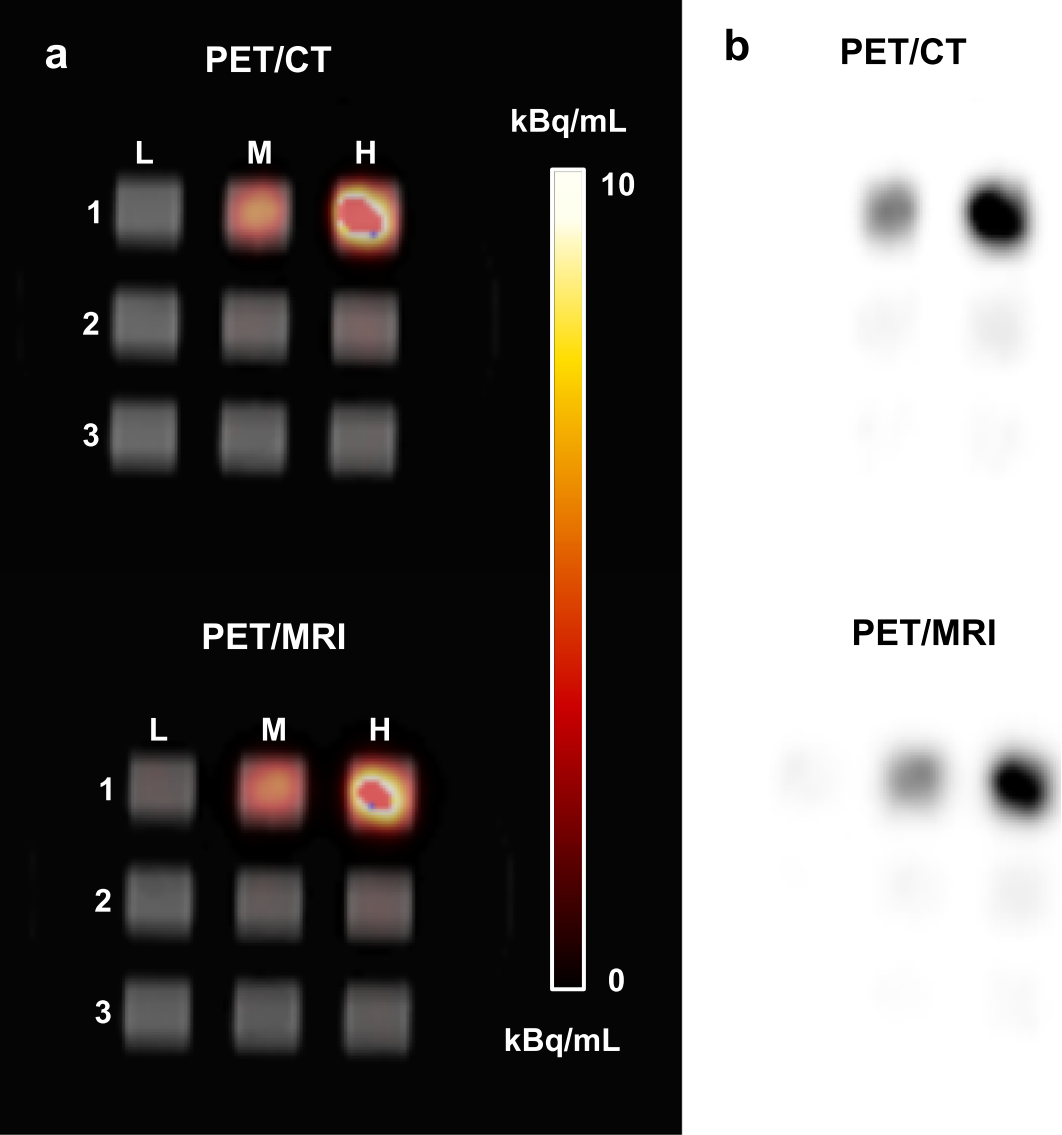
PET images of 3 × 3 cubic-well plate containing ^89^Zr-labeled Jurkat cells suspended in Geltrex™ matrix in a 1-cm^3^ cube acquired using PET/CT and PET/MRI together with their **a** corresponding co-registered CT image and **b** in greyscale. Cell numbers and specific activities per well (kBq/10^6^ cells; indicated in parenthesis) were as follows: L1: 1.07 × 10^6^ (0.67). L2: 1.50 × 10^5^ (0.42). L3: 6.70 × 10^4^ (0.14). M1: 8.20 × 10^5^ (7.23). M2: 3.25 × 10^4^ (15.41). M3: 3.61 × 10^4^ (6.61). H1: 3.99 × 10^5^ (30.63). H2: 1.21 × 10^5^ (8.47). H3: 6.20 × 10^4^ (6.60)

Figure 4 illustrates representative coronal PET images of 6-well plates with different numbers of ^89^Zr-labeled cells and specific activities per well. In general, lower cell numbers with a higher specific activity could be detected with a higher contrast-to-noise ratio compared to wells with a higher cell number and a lower specific activity. In the example images shown, 5.2 × 10^5^ cells with a specific activity of 2.2 kBq/10^6^ cells could be clearly visualized (Fig. 4, A2), whereas the lowest cell number that could be detected was 6.8 × 10^4^, when the specific activity was 27.8 kBq/10^6^ cells (Fig. 4, B2). Similarly, 1.1 × 10^6^ cells with a specific activity of 0.7 kBq/10^6^ cells could not be clearly visualized on PET images of the 3 × 3 cubic well structure (Fig. 5, L1) while 3.3 × 10^4^ cells could be detected when the specific activity was 15.4 kBq/10^6^ cells (Fig. 5, M2).

The theoretical lower limit of detection (LLD) for PET/CT and PET/MRI, together with the specific activity at this LLD, is given in Table 1. Detailed calculations for obtaining LLD are presented in Supporting Fig. S9. The calculations yielded a slightly lower LLD for PET/MRI compared to PET/CT where values were of the order of 10^5^ cells. The LLD values obtained for the 3 × 3 cubic-well plate were lower than those obtained for the 6-well experiment, which can be explained by the higher density in the 1-cm^3^ wells (Supporting Table S4).

**Table 1:** Theoretical lower limit of detection (LLD) for PET/CT and PET/MRI with respect to cell number and specific activity

	6-well plates		3 × 3 cubic-well	
	LLD_PET/MRI_	LLD_PET/CT_	LLD_PET/MRI_	LLD_PET/CT_
Cell number	5.24 × 10^5^ (1.05 × 10^4^)*	5.88 × 10^5^ (1.07 × 10^4^)*	4.56 × 10^5^ (2.84 × 10^3^)*	4.83 × 10^5^ (3.22 × 10^3^)*
Specific activity [kBq/10^6^ cells]	3.26 (0.07)*	3.72 (0.07)*	5.46 (0.03)*	5.25 (0.04)*

*Values in parentheses indicate the theoretical LLD values according to the Rose criterion (CNR = 5) as a threshold for detectability

**Fig. 6:**
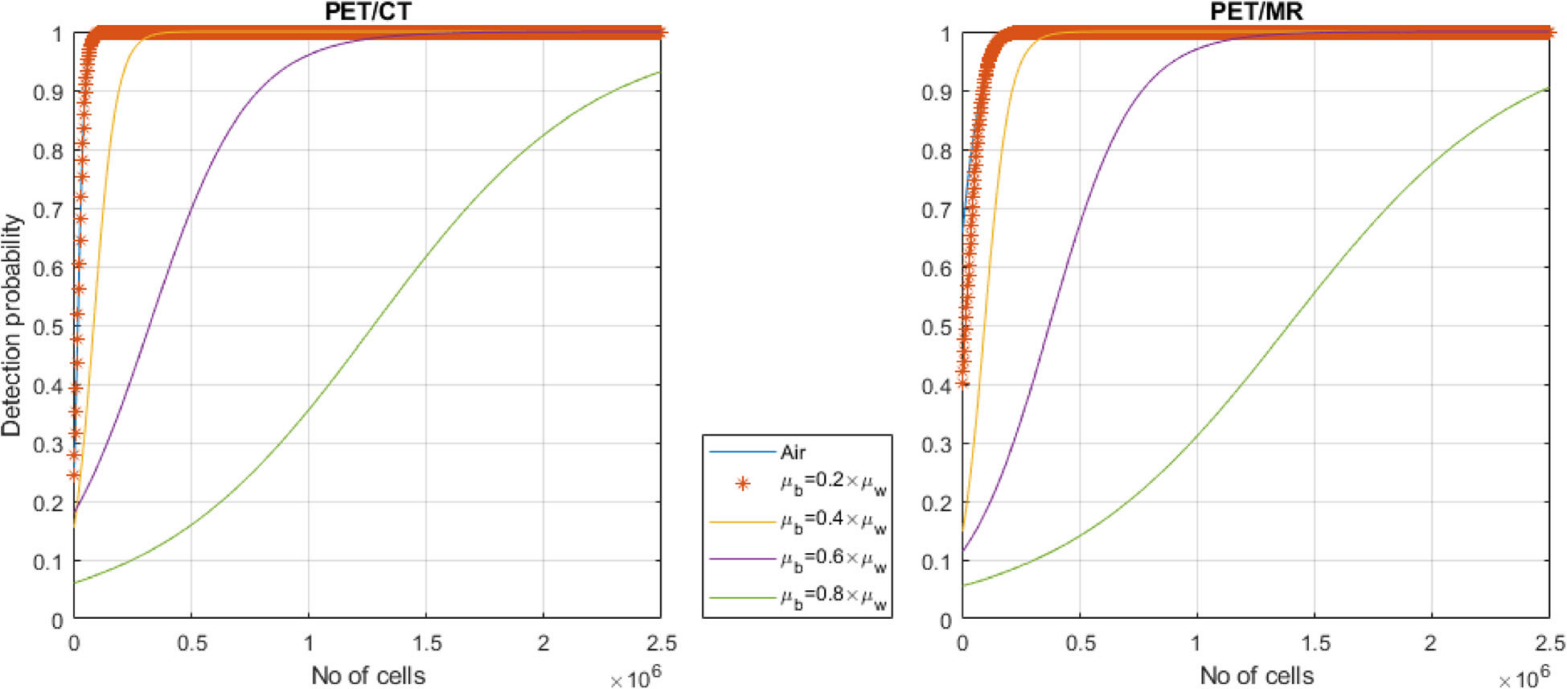
Detection probability of ^89^Zr-labeled cell suspensions at different background levels for **a** PET/CT and **b** PET/MRI. *μ*_w_, *μ*_b_: mean radioactivity concentration in ^89^Zr-containing wells and the background respectively

For the 6-well plates, plots of detection probability vs. cell number for both scanners are presented in Fig. 6. At the LLD, ^89^Zr-labeled cells could be detected with > 90% probability. As expected, a decrease in the detection probability of ^89^Zr-labeled cell suspensions was observed for increasing background levels and decreasing cell numbers per well. For a given detection probability and depending on the level of background activity, differences of two orders of magnitude could be observed in the minimum number of detectable ^89^Zr-labeled cells (Supporting Table S5).

**Fig. 7:**
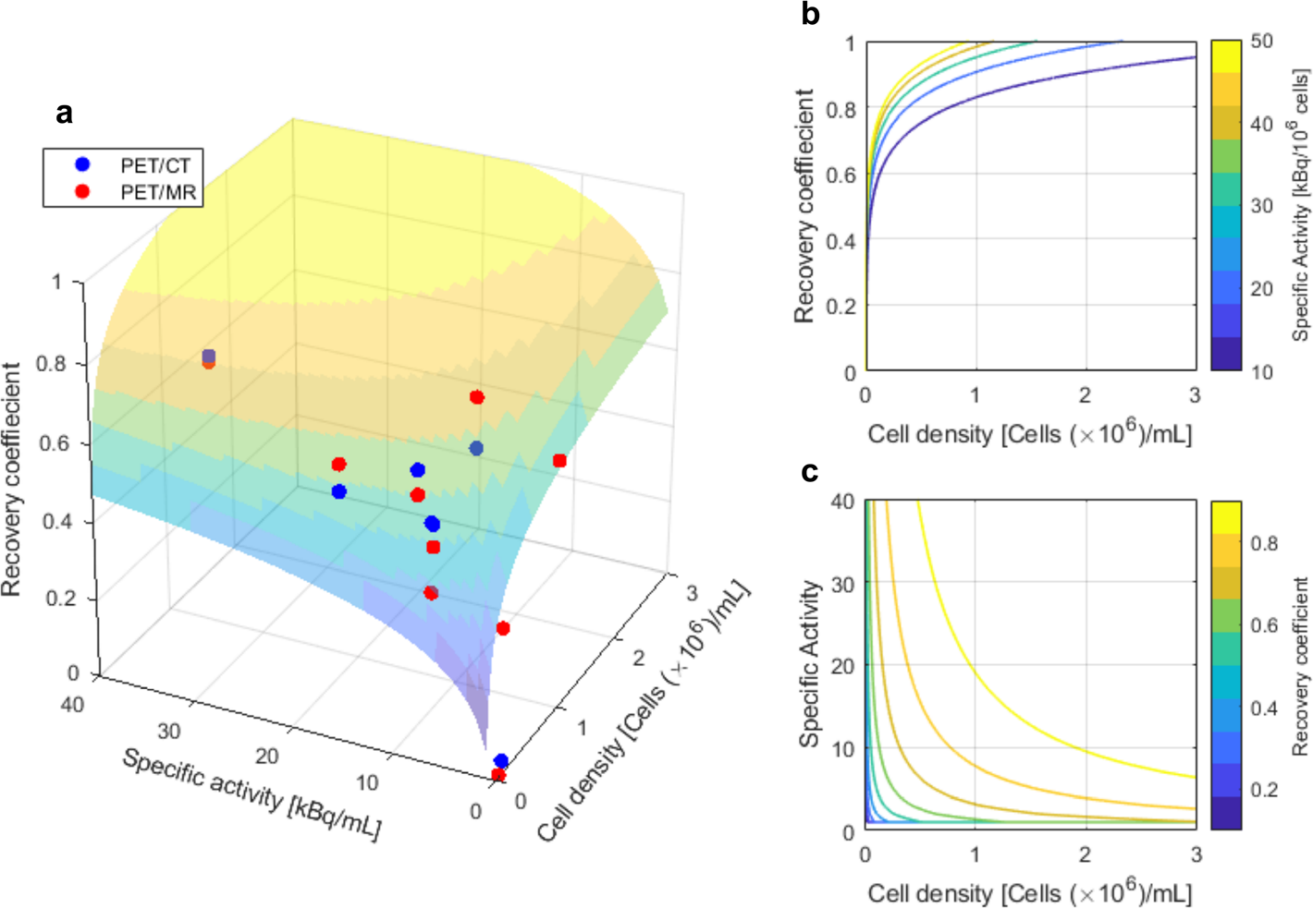
Recovery coefficient as a function of cell density (10^6^ cells/mL) and specific activity (kBq/10^6^ cells) for the 3 × 3 cubic-well plate. **a** Scatter plot of recovery coefficient vs. cell density and specific activity values and corresponding fit surface obtained through a logarithmic model function: *y* = 0.11 × log(CD × SA) + 0.57; CD = cell density; SA = specific activity. **b** Contour plot of specific activity as a function of recovery coefficient and cell density. The contours correspond to specific activities ranging from 10 to 50 kBq/10^6^cells. **c** Contour plot of recovery coefficient as a function of specific activity and cell density. Contour lines indicate recovery coefficients ranging from 0.1 to 1.0

Figure 7 presents the image recovery coefficient as a function of cell density and specific activity for the 3 × 3 cubic-well plate. In general, an improvement in contrast recovery was observed with increasing specific activity and cell density, indicating that the distribution of cells within a defined volume is an additional factor affecting detection. For the 3 × 3 cubic-well plate, convergence to a recovery coefficient of 1 for the specific activity obtained at LLD (~ 5.3 kBq/10^6^ cells for both scanners) would require ~ 8.8 × 10^6^ cells/mL.

## Discussion

Cell labeling and tracking is a rapidly growing area of clinical interest, particularly in the context of monitoring and evaluating novel cellular therapies (such as CAR-T cells). The sensitivity and quantification of cell labeling approaches are important for the successful clinical translation of this technology, but little research has been undertaken to evaluate this on a clinical scanner. [^89^Zr]Zr(oxinate)^4^ has been used as a direct cell labeling method for cellular tracking in preclinical models and holds great promise for clinical imaging due to the superior sensitivity offered by PET imaging compared to the conventional SPECT approach using [^111^In]In-oxine.

This is the first study to examine the theoretical detection limit and quantification of ^89^Zr-labeled cells on a human PET system with respect to therapeutic cell tracking. Using an in vitro approach, cell numbers in the magnitude of 10^5^ and 10^4^ with activity concentrations as low as 250 Bq/mL and 565 Bq/mL respectively could be detected during a 30-min scan. These activity concentrations are higher than those reported for [^18^F]-FDG labeled cells imaged in vitro on a clinical PET/CT, where activity concentrations as low as 50 Bq/mL could be visualized during a 5 min acquisition. This difference can in part be attributed to the much higher positron decay probability of fluorine-18 (96.9%) compared to zirconium-89 (22.7%), with another factor being the emission of coincident non-annihilation photons for zirconium-89, which leads to photon events that reduce image contrast. However, a major advantage of zirconium-89 over fluorine-18 is its much longer half-life (*t*_1/2_ = 78.4 h vs. 109 min), which is a requirement for longitudinal cell tracking over several days. Furthermore, zirconium-89 is a residualizing isotope, with intracellular binding to nuclear and cytoplasmic proteins ensuring retention inside the cell.

During a typical clinical white blood scintigraphy procedure, 10.0–18.5 MBq of ^111^In-labeled leucocytes are injected into patients to track cells and image sites of inflammation and infection [22]. Tumor-infiltrating lymphocytes and peripheral blood mononuclear cells could be tracked and imaged in patients with a maximum of 1.3–3.7 kBq/10^6^ cells with the corresponding range of injected number of cells being 1.5–9.2 × 10^9^ [3, 6]. This specific activity corresponds to the lowest specific activity of ^89^Zr-labeled cells in this study, where cells in the magnitude of 10^5^ could still be detected. With its superior sensitivity over SPECT, PET has the potential to detect cells labeled with less activity or alternatively a smaller number of cells. Preclinically, ^89^Zr-labeled cells have been tracked with PET at approximately 10 times lower activities compared to those labeled with Indium-111 [23]. Recent developments in the whole-body PET scanner technology offer the potential of sensitivity to increase by a further 40-fold for the whole body and 4–5-fold for a single organ [24]. Compared to conventional PET, this could allow the use of lower cellular activities or even fewer cells could be detected within the organ of interest.

Given the radiosensitivity of lymphocytes and their subsets, we examined the lower end of the radioactivity spectrum associated with cells for imaging purposes that have been shown not to cause significant cell damage. In preclinical animal models, the damage to γδ-T cells was minimal at specific activities up to 20 kBq/10^6^ cells [13], whereas CAR-T cells maintained their functionality and viability up to 70 kBq/10^6^ cells [21] and ^89^Zr-labeled bone marrow cells were not altered at 16.6 kBq/10^6^ cells and could be imaged up to 7 days after injection in vivo [11]. Therefore, our focus in this study was to quantify the lower limits of cell detection with specific activities of up to ~ 35 kBq/10^6^ cells. Our data suggests that cells with specific activities as low as 0.7–2.2 kBq/10^6^ cells can still be visualized on a human PET scanner which is promising for the potential use of ^89^Zr-labeled cells in vivo.

The specific activity per cell is one of the major limiting factors for cellular imaging using radioactive labeling approaches, given that labeling and tracking must not significantly alter the phenotype, viability, and functionality of labeled cells. Ideally, the radioactivity per cell should not affect any of these characteristics but at the same time should be sufficient to allow the visualization of labeled cells on imaging. These characteristics are pivotal prerequisites that need to be incorporated into all in vivo cell tracking and especially clinical imaging scenarios.

We have shown that detectability, as measured by the contrast-to-noise ratio, is dependent on the total cellular activity, which is the product of cell number and the specific activity (activity/10^6^ cells). Figure 4 demonstrates the strong interplay between the cell number and specific activity for cell detection in wells. While 6.1 × 10^4^ cells with a specific activity of 6.9 kBq/10^6^ cells (C3) were not visible on imaging, a similar number of cells (6.8 × 10^4^) could be detected with a 4-times higher specific activity (B2). Likewise, 1.7 × 10^5^ cells could not be detected (C2) compared to 5.2 × 10^5^ cells (A2) with a similar specific activity. Similarly, Fig. 5 shows that 3.3 × 10^4^ cells with a specific activity of 15.4 kBq/10^6^ cells could just be visualized (M2) while 3.6 × 10^4^ cells carrying less than half the specific activity could not be detected (M3).

The limitations of this work include that the imaged cell suspensions in each well are not a true representation of an in vivo cellular infiltration. Images revealed that cells tended to coalesce into “hot spots” within the wells. Dispersal circulation and infiltration in vivo may result in less dense accumulation, a feature which will reduce detectability. As illustrated in Fig. 7, the ability to visualize ^89^Zr-labelled cell deposits will not only be influenced by the specific activity on cells and cell number, but also the distribution and density of cells within the target region. The effect of cell density on detectability can be observed in Fig. 5, where wells with similar specific activities (7.2 kBq/10^6^, M1 vs. 6.6 kBq/10^6^, M3) could and could not be visualized respectively, owing to differences in cell density (8.2 × 10^5^ cells/mL, M1 vs. 3.6 × 10^4^ cells//mL, M3).

Similarly, we have not accounted for dilution of the radioactive signal over time due to cell proliferation as the plates were only imaged once directly after labeling. Signal dilution could significantly affect in vivo detection at later time points. Imaging 7 days after injection of ^89^Zr-labeled cells results in an approximately 25% decrease of total cellular activity due to radioactive decay alone, without accounting for additional effects of label dilution due to cellular proliferation.

Our measured data also did not account for non-specific radioactivity, which will decrease the contrast-to-noise ratio and therefore the detectability. As a consequence, our measured data shows unusually high CNRs that would normally not be observed on patient images. Cells labeled with [^89^Zr]Zr-(oxinate)_4_ cannot be distinguished from dead cells, and the leakage of activity from dead cells is likely to account for non-specific activity in neighboring tissues and bone over time due to the bone-seeking properties of Zirconium-89. The detection limit of ^89^Zr-labeled cells is expected to be higher for in vivo cell tracking, because of non-specific signal, higher photon attenuation, and a higher level of scatter and random coincidences potentially reducing contrast and increasing image noise. In an attempt to model the effect of non-specific radioactivity on detection, simulations for a non-specific background signal were performed. As expected, a decrease in detection probability was observed with increasing background levels. The number of cells required to achieve a detection probability of 90% increased by approximately two orders of magnitude for backgrounds ranging from 10 to 80% of the radioactivity concentration in wells. Taken together, the results provide a best-case scenario for the in vivo detection of ^89^Zr-labeled cells on clinical PET systems.

## Conclusion

[^89^Zr]Zr(Oxinate)_4_ offers a promising solution to the emerging need for a long-lived PET labeling approach for in vivo cell tracking. This study shows the feasibility of detecting ^89^Zr-labeled cells on human PET/CT and PET/MRI scanners and presents detection limit data that could be used to guide in vivo cell tracking studies using [^89^Zr]Zr(oxinate)_4_ in the future. This work has important implications for human cell labeling procedures, which require the detection of low cell numbers while minimizing radiation dose per cell, particularly when using radiosensitive cells such as T lymphocytes or CAR T cells.

## Supplementary information

**Additional file 1: Table S1.** Radiochemical yield for the formation of [89Zr]Zr(Oxinate)4 using 6.4-7.5 MBq [89Zr]Zr-Oxalate with increasing amount of Oxine (8-hydroxycholin) as ligand to optimize the content of Oxine in the reaction solution (n=3). Radiochemical yield was measured by iTLC. **Figure S1.** Representative radio iTLC chromatogram of [89Zr]Zr-(Oxinate)4 (Rf ~ 0.9). **Figure S2.** Representative radio iTLC chromatogram of [89Zr]Zr-Oxalate (Rf ~ 0). **Figure S3.** Cellular uptake of [89Zr]Zr-Oxalate into Jurkat cells at different incubation conditions. **Table S2.** Cellular conditions for the 6-well plate experiment. **Table S3.** Cellular conditions for the 3D structure experiment. **Figure S4.** Experimental design for PET scanning. **Figure S5.** Mean viability of unlabeled cells as control compared to the mean viability of cells after labelling with [89Zr]Zr(oxinate)4. **Figure S6.** Pearson correlation between the overall contrast-to-noise ratio (CNR) and specific activity [kBq/106 cells] as obtained from PET/CT and PET/MR images. **Figure S7.** Pearson correlation between the cell number [106 cells] and the respective contrast-to-noise ratio (CNR) per well acquired using PET/CT and PET/MRI. **Figure S8-1.** 6-well plate experiment. **Figure S8-2.** 3×3 cubic-well plate. **Figure S9-1.** Linear regressions of contrast-to-noise ratio (CNR) versus specific activity (a, c) and specific activity versus cell number (b, d) for both PET/CT (a, b) and PET/MRI (c, d) for the 6-well plate experiment. **Figure S9-2.** Linear regressions of contrast-to-noise ratio (CNR) versus specific activity (a, c) and specific activity versus cell number (b, d) for both PET/CT (a, b) and PET/MRI (c, d) for the 3×3 cubic-well plate. **Table S4.** Well cell density (106 cells/mL) in wells for 6-well plates and the 3×3 cubic-well plate. Data are presented as median [range]. a Mann-Whitney U test. **Table S5.** Detection probability calculations for the 6-well plate experiment.

## Data Availability

The datasets used from this study can be made available from the corresponding author on reasonable request.
