# Supplementary material for: Detection limit of 89Zr-labeled T cells for cellular tracking: an in vitro imaging approach using clinical PET/CT and PET/MRI
Source: EJNMMI Res. 2020 Jul 14;10:82. doi: 10.1186/s13550-020-00667-5 (PMC7360010; doi:10.1186/s13550-020-00667-5)
Supplement: Supplementary file 1 — Additional file 1: Table S1. Radiochemical yield for the formation of [89Zr]Zr(Oxinate)4 using 6.4-7.5 MBq [89Zr]Zr-Oxalate with increasing amount of Oxine (8-hydroxycholin) as ligand to optimize the content of Oxine in the reaction solution (n=3). Radiochemical yield was measured by iTLC. Figure S1. Representative radio iTLC chromatogram of [89Zr]Zr-(Oxinate)4 (Rf ~ 0.9). Figure S2. Representative radio iTLC chromatogram of [89Zr]Zr-Oxalate (Rf ~ 0). Figure S3. Cellular uptake of [89Zr]Zr-Oxalate into Jurkat cells at different incubation conditions. Table S2. Cellular conditions for the 6-well plate experiment. Table S3. Cellular conditions for the 3D structure experiment. Figure S4. Experimental design for PET scanning. Figure S5. Mean viability of unlabeled cells as control compared to the mean viability of cells after labelling with [89Zr]Zr(oxinate)4. Figure S6. Pearson correlation between the overall contrast-to-noise ratio (CNR) and specific activity [kBq/106 cells] as obtained from PET/CT and PET/MR images. Figure S7. Pearson correlation between the cell number [106 cells] and the respective contrast-to-noise ratio (CNR) per well acquired using PET/CT and PET/MRI. Figure S8-1. 6-well plate experiment. Figure S8-2. 3×3 cubic-well plate. Figure S9-1. Linear regressions of contrast-to-noise ratio (CNR) versus specific activity (a, c) and specific activity versus cell number (b, d) for both PET/CT (a, b) and PET/MRI (c, d) for the 6-well plate experiment. Figure S9-2. Linear regressions of contrast-to-noise ratio (CNR) versus specific activity (a, c) and specific activity versus cell number (b, d) for both PET/CT (a, b) and PET/MRI (c, d) for the 3×3 cubic-well plate. Table S4. Well cell density (106 cells/mL) in wells for 6-well plates and the 3×3 cubic-well plate. Data are presented as median [range]. a Mann-Whitney U test. Table S5. Detection probability calculations for the 6-well plate experiment. [file 13550_2020_667_MOESM1_ESM.docx]

**Supporting information**

**Title:** Detection limit of ^89^Zr-labeled T-cells for cellular tracking: an *in vitro* imaging approach using clinical PET/CT and PET/MRI

**European Journal of Nuclear Medicine and Molecular Imaging research**

**(EJNMMI research)**

**Authors:** Laura M Lechermann^1,2^, Roido Manavaki^1^, Bala Attili^1,2^, Doreen Lau^1,2^, Lorna B Jarvis^4^, Tim D Fryer^3,4^, Nick Bird^5^, Luigi Aloj^1,5^, Neel Patel^3^, Bristi Basu^2,7^, Matthew Cleveland^6^, Franklin I Aigbirhio^3,4^, Joanne L Jones^4^ and Ferdia A Gallagher^1,2^

**Affiliations**

^1^ Department of Radiology, University of Cambridge, Cambridge, UK

^2^ Cancer Research UK Cambridge Institute, University of Cambridge, Cambridge, UK

^3^ Wolfson Brain Imaging Centre, University of Cambridge, Cambridge, UK

^4^ Department of Clinical Neurosciences, University of Cambridge, Cambridge, UK

^5^ Department of Nuclear Medicine, Addenbrooke’s Hospital, Cambridge, UK

^6^ GSK Medicines Research Centre, Gunnels Wood Road, Stevenage, UK

^7^ Department of Oncology, University of Cambridge, Cambridge, UK

Correspondence to:	Dr Ferdia A Gallagher ([fag1000@cam.ac.uk](mailto:fag1000@cam.ac.uk))

Laura Lechermann ([lmtl2@cam.ac.uk](mailto:lmtl2@cam.ac.uk))

**Contents:**

1. [^89^Zr]Zr(oxinate)_4_ synthesis and optimization of [^89^Zr]Zr(oxinate)_4_ synthesis for Oxine content – **Table S1**.....................................................................................................4
2. iTLC chromatogram of [^89^Zr]Zr(oxinate)_4_ and [^89^Zr]Zr-Oxalate – **Fig. S1** and **S2**......5
3. Uptake of free ‘^89^Zr^4+^’ into Jurkat cells under different conditions – Fig. S3..............7
4. Detection limit experiment – experimental procedure................................................8
5. PET/CT and PET/MRI acquisition – **Fig. S4**.............................................................13
6. Well plate attenuation map generation.....................................................................15
7. Viability of unlabeled cells versus labeled cells after each scan (6-well plates) and versus labeled cells before the scan (3D structure) – **Fig. S5**..................................16
8. Overall correlation between specific activity on cells and the contrast-to-noise ratio on PET/CT and PET/MRI images – **Fig. S6**.............................................................17
9. Overall correlation between the cell number and the contrast-to-noise ratio on PET/CT and PET/MRI images – **Fig. S7**..................................................................18
10. Correlation and Bland-Altman analysis of CNRs and total
    cellular activities obtained PET/CT versus PET/MRI – **Fig. S8**...........................19
11. Calculation of theoretical lowest limit of detection (LLD) for
    PET/CT and PET/MRI – **Fig. S9**..........................................................................21
12. Estimation of cell densities – **Table S4**................................................................23
13. Detection probability calculations – **Table S5**......................................................24

**Abbreviations**

Bkg	Background

Complete RPMI	RPMI medium containing 10 % FBS, 10 mM HEPES and 200 U/l penicillin, 0.1 g/l streptomycin

CT	Computed Tomography

EtOAc	Ethyl acetate

iTLC	instant thin layer chromatography

kBq	kilobequerel

mm	millimeter

M	Molarity

PBS	Phosphate buffered saline

PET	Positron Emission Tomography

1. **[^89^Zr]Zr(oxinate)_4_** **synthesis and optimization for Oxine content**

Zirconium-89 as Zr^4+^ in 1 M oxalic acid was buffered to pH 7 with 1 M Na_2_CO_3_ and diluted to 500 μL with water. This neutralized [^89^Zr]Zr-oxalate stock solution was used for radiotracer synthesis and also as the control for the *in vitro* cell labeling experiments. In order to minimize the exposure and uptake of free oxine, the amount of ligand (oxine) for the synthesis of [^89^Zr]Zr(oxinate)_4_ was optimized for the amount of activity used in this experimental procedure (**Table S1**). 250 μL of a 1 mg/mL 8-hydroxychinoline (oxine) solution in 0.1 M 4-(2-hydroxyethyl)-1-piperazineethanesulfonic acid (HEPES) was added to 6.4-7.5 MBq of neutralized [^89^Zr]Zr-Oxalate (pH 7). The solution was incubated for 30 min at room temperature before being used without further purification for cell labelling.

| **Amount of Oxine [μg]** | **Radiochemical yield [%]** |
| --- | --- |
| 10 | < 1 % |
| 100 | 56.6 ± 0.2 |
| 200 | 83.2 ± 4.5 |
| 250 | 94.5 ± 2.9 |

**Table S1** Radiochemical yield for the formation of [^89^Zr]Zr(Oxinate)_4_ using 6.4-7.5 MBq [^89^Zr]Zr-Oxalate with increasing amount of Oxine (8-hydroxycholin) as ligand to optimize the content of Oxine in the reaction solution (n=3). Radiochemical yield was measured by iTLC

2. **iTLC chromatogram of [^89^Zr]Zr(Oxinate)_4_ and [^89^Zr]Zr-Oxalate**

**Fig. S1 Representative radio iTLC chromatogram of [^89^Zr]Zr-(Oxinate)_4_ (R_f_ ~ 0.9).**

The reaction solution containing [^89^Zr]Zr(Oxinate)_4_ was spotted onto a ca. 9 cm long iTLC plate at 1 cm from the bottom of the plate (starting line), dried at room temperature and was then put into a TLC chamber to develop in 100% EtOAc as mobile phase. [^89^Zr]Zr(Oxinate)_4_ moves with the EtOAc whereas free ^89^Zr^4+^ in 1 M oxalic acid stays at the origin. iTLC plates were analyzed on a radio-TLC plate reader equipped with a PET detector using Laura software (Lablogic, UK).

**Fig. S2 Representative radio iTLC chromatogram of [^89^Zr]Zr-Oxalate (R_f_ ~ 0).**
^89^Zr^4+^ in 1 M oxalic acid, [^89^Zr]Zr-Oxalate, from the created stock solution was spotted onto a ca. 9 cm long iTLC plate at 1 cm from the bottom of the plate (starting line), dried at room temperature and was then put into a TLC chamber to develop in 100% EtOAc as mobile phase. Free ^89^Zr^4+^ in 1 M oxalic stays at the origin and does not move with EtOAc.

3. **Uptake of [^89^Zr]Zr-Oxalate as free ^89^Zr^4+^ into Jurkat cells**

150-300 kBq of free neutralized [^89^Zr]Zr-oxalate solution was added to Jurkat cells in either PBS or complete RPMI medium and incubated for 30 and 60 min as the control for *in vitro* cell labeling experiments. ^89^Zr]Zr-oxalate showed no significant uptake into Jurkat T-cells (< 1% of the added activity). **Fig. S3** provides [^89^Zr]Zr-Oxalate uptake values at different incubation conditions.

**Fig. S3 Cellular uptake of [^89^Zr]Zr-Oxalate into Jurkat cells at different incubation conditions**

**(a)** 30 min incubation with increasing amount of activity added to Jurkat cells (n=3)

**(b)** constant (average) activity added with increasing incubation time (n=3)

4. **Detection limit experiment – Experimental procedure**

*6-well plate experiment*

After Jurkat cells were harvested from 75 cm^3^ flasks and counted, 3-5 x 10^6^ Jurkat cells in 1.5 mL PBS or complete RPMI medium were labelled with ^89^Zr]Zr(oxinate)_4_ at room temperature for 30 min in a scintillation vial ①. ^89^Zr-labeled cells were centrifuged (5 min, 500 g) and washed twice with PBS. The pellet was resuspended in 0.5 mL complete RPMI medium measured in a well counter to calculate the uptake efficacy [%] ②. This cell suspension was used to distribute ^89^Zr-labeled cells into 3 wells of a 6-well plate at different cell concentrations ③. A total volume of 2mL per well was reached by adding completed RPMI medium to the well containing ^89^Zr-labeled cells. For PET imaging, two 6-well plates containing ^89^Zr-labeled cell suspensions were placed next to each other on a 1 cm thick polystyrene sheet to be scanned first on a clinical PET/CT scanner for 30 min ④ and subsequently on a clinical PET/MRI scanner for 30 min ⑤. Directly after PET/CT and PET/MR imaging, the cell suspension in each well was transferred to a 2 mL Eppendorf tube, centrifuged (5 min, 500 g) to form a pellet, resuspended in 0.5 mL complete RPMI medium and counted in the well counter for cellular activity retention ⑥. Cells were counted in a hemocytometer and cell viability was quantified as a last step for each cell suspension per well ⑦.

| **Plate** | **Well** | **Cell number [10^6^ cells]** | **Cellular activity (well counter) [kBq]** | **Specific activity [kBq/10^6^ cells]** | **Cellular activity (PET/CT) [kBq]** | **Cellular activity (PET/MRI) [kBq]** |
| --- | --- | --- | --- | --- | --- | --- |
| **1** | 10^6^ | 1.58 | 7.72 | 4.89 | 8.51 | 10.68 |
|  | 10^5^ | 0.14 | 1.26 | 9.02 | 1.32 | 1.64 |
|  | 10^4^ | 0.03 | 0.10 | 4.07 | 0.03 | 0.22 |
| **2** | 10^6^ | 1.28 | 3.41 | 2.66 | **n/a** | 3.81 |
|  | 10^5^ | 0.06 | 0.03 | 0.43 | **n/a** | 0.04 |
|  | 10^4^ | 0.03 | 0.02 | 0.63 | **n/a** | 0.01 |
| **3** | 10^6^ | 0.24 | 2.25 | 9.23 | 4.87 | 5.46 |
|  | 10^5^ | 0.03 | 0.19 | 7.60 | 0.32 | 0.35 |
|  | 10^4^ | **n/a** | **n/a** | **n/a** | **n/a** | **n/a** |
| **4** | 10^6^ | 0.24 | 2.27 | 9.38 | 4.73 | 4.93 |
|  | 10^5^ | 0.01 | 0.09 | 8.82 | 0.37 | 0.44 |
|  | 10^4^ | **n/a** | **n/a** | **n/a** | **n/a** | **n/a** |
| **5** | 10^6^ | 0.38 | 12.22 | 31.91 | 22.00 | 24.47 |
|  | 10^5^ | 0.07 | 1.87 | 27.77 | 2.95 | 3.23 |
|  | 10^4^ | 0.01 | 0.18 | 18.37 | 0.01 | 0.32 |
| **6** | 10^6^ | 0.27 | **n/a** | **n/a** | 19.78 | 21.67 |
|  | 10^5^ | 0.15 | **n/a** | **n/a** | 3.38 | 3.78 |
|  | 10^4^ | 0.05 | **n/a** | **n/a** | 0.01 | 0.34 |
| **7** | 10^6^ | 0.85 | 6.62 | 7.83 | 11.60 | 12.30 |
|  | 10^5^ | 0.17 | 0.59 | 3.57 | 0.90 | 1.00 |
|  | 10^4^ | 0.06 | 0.42 | 6.93 | 0.50 | 0.30 |
| **8** | 10^6^ | 0.90 | 5.74 | 6.41 | 13.30 | 13.30 |
|  | 10^5^ | 0.14 | 0.65 | 4.71 | 0.90 | 0.80 |
|  | 10^4^ | 0.22 | 0.35 | 1.57 | 0.40 | 0.10 |
| **9** | 10^6^ | 2.17 | 4.02 | 1.85 | 4.20 | 4.60 |
|  | 10^5^ | 0.55 | 1.19 | 2.16 | 1.20 | 1.40 |
|  | 10^4^ | 0.16 | 0.23 | 1.42 | 0.20 | 0.30 |
| **10** | 10^6^ | 1.36 | 3.67 | 2.70 | 3.80 | 4.20 |
|  | 10^5^ | 0.52 | 1.13 | 2.18 | 1.10 | 1.20 |
|  | 10^4^ | 0.15 | 0.23 | 1.57 | 0.10 | 0.20 |
| **11** | 10^6^ | 1.78 | 15.57 | 8.75 | 10.60 | 11.30 |
|  | 10^5^ | 0.62 | 6.45 | 10.41 | 3.70 | 4.00 |
|  | 10^4^ | 0.11 | 2.48 | 21.96 | 1.40 | 1.80 |
| **12** | 10^6^ | 1.26 | 14.03 | 11.14 | 10.60 | 11.30 |
|  | 10^5^ | 0.55 | 5.80 | 10.54 | 3.90 | 4.10 |
|  | 10^4^ | 0.19 | 1.56 | 8.27 | 0.90 | 0.90 |

**Table S2 Cellular conditions for the 6-well plate experiment:** cell number, total cellular activity as measured by the well counter, specific activity (based on the total number divided by the total cellular activity as measured in the well counter) and cellular activities per well obtained on PET/CT and PET/MRI.

*3×3-well array with 1-cm^3^ cubic wells*

After Jurkat cells were harvested from 75 cm^3^ flasks and counted, three times 3 x 10^6^ Jurkat cells in 1.5 mL PBS or complete RPMI medium were prepared to be labelled separately with ^89^Zr]Zr(oxinate)_4_ at room temperature for 30 min in a scintillation vial ① to generate cell suspensions with low (L), medium (M) and high (H) specific activities. ^89^Zr-labeled cells were centrifuged (5 min, 500 g) and washed twice with PBS. The pellet was resuspended in 0.5 mL complete RPMI medium and measured in a well counter to calculate the uptake efficacy [%]. The labelled cell suspension in each vial was transferred to a 2 mL Eppendorf tube, centrifuged (5 min, 500 g) to form a pellet, resuspended in 0.5 mL complete RPMI medium and counted in the well counter for total cellular activity retention. From each of the labelled cell suspensions different fractions (cell numbers ranging from 10^4^ - 10^6^) ② were taken an put into separate 1.5 mL Eppendorf vial, filled up to 0.5 mL with complete RPMI medium and counted again in the well counter for total cell associated activity ③. Cells were counted in a hemocytometer and cell viability was quantified for each cell suspension ④ before being mixed with 0.5 mL Geltrex™ matrix in the same vial which was kept on ice (ca. 4 ºC). The ice cold ^89^Zr-labeled cell suspensions mixed with Geltrex™ were transferred into the 1 cm^3^ cubes of the 3D printed structure ⑤ which was immediately put into an incubator at 37 ºC for 30 min with a petri dish lid loosely covering the top of the structure so that the Geltrex™-cell suspension mixture could solidify. For PET imaging, the 3D structure was put into a petri dish, covered with a lid and placed on a 1 cm thick polystyrene sheet and scanned first on a clinical PET/CT scanner for 30 min ⑥ and subsequently on a clinical PET/MRI scanner for 30 min ⑦.

| **Cube** | **Cell number [10^6^ cells]** | **Cellular activity (well counter) [kBq]** | **Specific activity [kBq/10^6^ cells]** | **Cellular activity (PET/CT) [kBq]** | **Cellular activity (PET/MRI) [kBq]** | **Recovery**  **Coefficient**  **(PET/CT)** | **Recovery**  **Coefficient**  **(PET/MR)** |
| --- | --- | --- | --- | --- | --- | --- | --- |
| **L-10^6^** | 1.07 | 0.72 | 0.67 | 0.01 | 0.45 | 0.01 | 0.01 |
| **L-10^5^** | 0.15 | 0.06 | 0.42 | 0.00 | 0.03 | 0.02 | 0.06 |
| **L-10^4^** | 0.07 | 0.01 | 0.14 | 0.00 | 0.00 | 0 | 0 |
| **M-10^6^** | 0.82 | 5.93 | 7.23 | 3.87 | 4.62 | 0.65 | 0.78 |
| **M-10^5^** | 0.03 | 0.50 | 15.41 | 0.32 | 0.36 | 0.63 | 0.69 |
| **M-10^4^** | 0.04 | 0.24 | 6.61 | 0.15 | 0.11 | 0.60 | 0.43 |
| **H-10^6^** | 0.40 | 12.22 | 30.63 | 9.95 | 9.77 | 0.81 | 0.80 |
| **H-10^5^** | 0.12 | 1.03 | 8.47 | 0.73 | 0.67 | 0.70 | 0.65 |
| **H-10^4^** | 0.06 | 0.41 | 6.60 | 0.24 | 0.23 | 0.59 | 0.54 |

**Table S3 Cellular conditions for the 3D structure experiment**: Cell number, total cellular activity as measured by the well counter, specific activity (based on the total number divided by the total cellular activity as measured in the well counter), cellular activities per 1 cm^3^ cube obtained on PET/CT and PET/MRI and the recovery coefficient for both PET/CT and PET/MRI.

5. **PET/CT and PET/MRI acquisition**

Two 6-well plates were scanned next to each other on a 1 cm thick polystyrene sheet containing 0.6 mm diameter spherical oil capsules visible on both the CT and MR images. The oil capsules were placed at the four corners of each well-plate to enable co-registration between PET/CT and PET/MR, facilitating CT-based attenuation correction of the PET data acquired on the PET/MR.

***PET/CT***: PET/CT imaging was performed with the plates placed centrally in the field-of-view (FOV). Following a CT for attenuation correction, a 30 minutes PET acquisition was performed. Emission data were reconstructed into a 256×256×47 matrix with 1.2×1.2×3.27 mm voxels, using time-of-flight ordered-subsets expectation-maximization (TOF-OSEM) with 4 iterations and 24 subsets. Corrections for normalization, dead-time, random events, scatter, attenuation, decay and sensitivity were applied as implemented on the scanner, together with an isotropic 2 mm full-width-at-half-maximum (FWHM) Gaussian filter post reconstruction. The CT acquisition parameters were as follows: tube voltage: 120 kV; tube current: 50 mA; rotation time: 0.8 s; spiral pitch factor: 1.375, matrix: 512×512; pixel size: 0.5×0.5 mm; slice thickness: 3.75 mm (reconstructed to 3.27 mm).

***PET/MRI***: After the PET/CT scan, the polystyrene support and well-plates were transferred for imaging on a GE SIGNA PET/MRI scanner. MR acquisition involved the manufacturer’s 2-point Dixon sequence. To ensure a fair comparison against PET/CT, emission data were acquired for 30 minutes divided by the radioactive decay factor between PET/CT and PET/MR acquisitions (i.e. >30 minutes). Images were reconstructed using TOF-OSEM with 6 iterations and 16 subsets, and an isotropic 2 mm FWHM Gaussian post-reconstruction filter. To approximately match the voxel size used for reconstruction of the PET data on PET/CT, a 256×256×89 matrix with 1.4×1.4×2.78 mm voxels was utilized. Corrections for normalization, deadtime, random events, scatter, decay and sensitivity were applied as implemented on the scanner. Attenuation correction of the PET data from the PET/MRI acquisitions used the CT images from the corresponding PET/CT acquisition for each experiment to generate attenuation maps for the well-plates [1].

1 cm^3^

Oil capsules

**a**

**b**

Polystyrene support

**Fig. S4 Experimental design for PET scanning:** (**a**) Two 6-well plates containing ^89^Zr-labeled cell suspensions in a total volume of 2 mL were placed next to each other on a 1-cm thick polystyrene sheet containing 0.6-mm diameter spherical oil capsules visible on both the CT and MR images to enable co-registration between PET/CT and PET/MR and facilitate CT-based attenuation correction of the PET data acquired on PET/MRI; (**b**) a 3D printed structure (5×5 cm, material: ClearVue™) containing nine 1 cm^3^ cubic wells with ^89^Zr-labeled Jurkat cells suspended in Geltrex™ matrix. The 3D structure was placed into a petri dish before being supported on the 1 cm thick polystyrene sheet containing 0.6-mm diameter spherical oil capsules. 3D printing of the cubic-well structure was performed at GlaxoSmithKline (Stevenage, UK) on a 3D Systems Viper printer using stereolithography technology.

6. **Well plate attenuation map generation**

In a first step and for each of the eight experiments, the CT component of the PET/CT acquisition was rigidly registered to the corresponding water or fat images automatically generated by the Dixon sequence, utilizing the oil capsules embedded in the polystyrene support as reference for co-registration. Image registration was performed manually in Analyze 12.0 (AnalyzeDirect Inc., Overland Park, KS, USA), and the quality of registrations was visually inspected.

Thresholding and manual editing were applied to the original CT image from the PET/CT scan to define a binary mask from the 6-well plates (i.e. excluding the scanner bed). This mask was subsequently multiplied with the CT to produce an image encompassing the well-plates only. The edited CT image of the well-plates was converted to a 511-keV attenuation map utilizing bilinear transformation [1] and transformed into the space of water/fat images using the rigid transformation parameters calculated in Analyze 12.0. To produce a complete attenuation map for attenuation correction on the PET/MRI scanner, the attenuation maps of the well plates were combined with attenuation templates of the objects present in the FOV of PET/MRI scanner during acquisition (bed cradle, plastic gap filler and posterior array) and smoothed with a 5 mm FWHM Gaussian filter. Well-plate attenuation map generation was performed using in-house software implemented in Matlab 2016b (Mathworks Inc, Nattick, NA, USA).

7. **Viability of unlabeled cells versus labeled cells after each
   scan (6-well plates) and versus labeled cells before the scan
   (3×3 cubic well plate)**

**Fig. S5 Mean viability of unlabeled cells as control compared to the mean viability of cells after labelling with [^89^Zr]Zr(oxinate)_4_. (a) 6-well plate experiment:** non-significant (p = 0.16) difference between the mean viability of unlabelled cells (73.0 % ± 16.1 %) labelled cells (69.2 % ± 18.0 %) after the end of all PET scans performed for the 6-well plates, **(b) 3×3 cubic-well plate experiment:** non-significant (p = 0.10) difference between the mean viability of unlabelled cells (75.2 % ± 3.0 %) and labelled cells (72.0 % ± 1.1 %) before the labelled cells were prepared in Geltrex™ matrix for subsequent imaging. Error bars indicate the standard deviation (SD) from the mean value; ns = not significant

8. **Overall correlation between specific activity on cells and the
    contrast-to-noise ratio on PET/CT and PET/MR images**

**Fig. S6 Pearson correlation between the overall contrast-to-noise ratio (CNR) and specific activity [kBq/10^6^ cells] as obtained from PET/CT and PET/MR images: (a)** for the 6-well plate experiment **(**PET/CT: r = 0.44, p = 0.02; PET/MRI: r = 0.49; p = 0.006), **(b)** for the **3×3 cubic-well plate**
**(**PET/CT: r = 0.85, p = 0.004; PET/MRI: r = 0.82; p = 0.007)

9. **Overall correlation between the cell number and the
    contrast-to-noise ratio on PET/CT and PET/MRI**

**Fig. S7 Pearson correlation between the cell number [10^6^ cells] and the respective contrast-to-noise ratio (CNR) per well acquired using PET/CT and PET/MRI**: **(a)** for the 6-well plate experiment (PET/CT: r=0.41, p=0.03; PET/MRI: r=0.42, p=0.02), **(b)** for the 3×3 cubic-well plate **(**PET/CT: r=0.79, p=0.52; PET/MRI: r=0.32, p=0.41)

10. **Correlation and Bland-Altman analysis of CNRs and total
    cellular activities obtained PET/CT versus PET/MRI**

**Fig. S8-1 6-well plate experiment:** Correlation (**a**) and Bland-Altman plot (**b**) of cellular activities [kBq] obtained by PET/CT imaging versus PET/MRI (r = 0.998, p<0.0001; Bland-Altman analysis: 0.93 bias (SD: 0.65), 95 % limit of agreement: -0.34-2.20) and Correlation (**c**) and Bland-Altman plot (**d**) of CNRs obtained by PET/CT imaging versus PET/MRI (p < 0.0001, r = 0.98, Bland-Altman analysis: 0.19 bias: (SD: 0.52), 95 % limits of agreement: 0.83-1.22)

**Fig. S8-2 3×3 cubic-well plate:** Correlation (**a**) and Bland-Altman plot (**b**) of cellular activities [kBq] obtained by PET/CT imaging versus PET/MRI (r = 0.992, p<0.0001; Bland-Altman analysis: 0.78 bias
(SD = 0.50), 95 % limit of agreement: -0.20-1.77) and Correlation (**c**) and Bland-Altman plot (**d**) of CNRs obtained by PET/CT imaging versus PET/MRI (p < 0.0001, r = 0.996, Bland-Altman analysis: 0.78 bias
(SD = 0.50), 95 % limits of agreement: -0.20-1.76)

11. **Calculation of theoretical lowest limit of detection (LLD) for
    PET/CT and PET/MRI**

**Fig. S9-1 Linear regressions of contrast-to-noise ratio (CNR) versus specific activity (a, c) and specific activity versus cell number (b, d) for both PET/CT (a, b) and PET/MRI (c, d) for the 6-well plate experiment.** Linear regression of specific activity vs. CNR used the upper 95% confidence interval (CI) of the x-intercept (=CNR). This value was subsequently inserted into the linear regression equation for CNR vs. cell number to derive the theoretical lower limit of detection (LLD). The obtained CNR, in the absence of background, was required to achieve the LLD

**Fig. S9-2 Linear regressions of contrast-to-noise ratio (CNR) versus specific activity (a, c) and specific activity versus cell number (b, d) for both PET/CT (a, b) and PET/MRI (c, d) for the 3×3 cubic-well plate.** Linear regression of specific activity vs. CNR used the upper 95% confidence interval (CI) of the x-intercept (=CNR). This value was subsequently inserted into the linear regression equation for CNR vs. cell number to derive the theoretical lower limit of detection (LLD). The obtained CNR, in the absence of background, was required to achieve the LLD

12. **Estimation of cell densities**

For the 1 cm^3^ cubic wells, cell density (10^6^ cells/mL) was calculated as the number of cells contained in the 1 mL well volume.

Due to the heterogeneity of cell distributions in the 6-well plates, cell density in each well was estimated by fitting the following polynomial equation to the PET image data:

$A_{ij}={a_{ij}CN}_{j}+{b_{ij}SA}_{j}+{c_{ij}(CN\cdot SA)}_{j}+d_{ij}+\varepsilon_{ij}$	(S1)

where for the *j*^th^ well when using scanner *i*=1 (PET/CT) or 2 (PET/MR), *A_ij_* is the well activity as measured on the PET images (kBq), *CN_j_* the cell number (×10^6^ cells), and *SA_j_* the specific activity (kBq/10^6^ cells). The model parameters are denoted by *a_ij_*, *b_ij_*, *c_ij_* and *d_ij_* and *ε*_ij_ are within-group (i.e. scanner) errors. Fitting was performed using a mixed effects model implemented in Matlab 2016b with fixed effects for the cell number and specific activity, and random effects accounting for differences between the two scanners.

The cell density in each voxel was calculated by substituting the voxel radioactivity concentration (kBq/mL) into equation S1 (term *A_ij_*) and solving for cell number per mL. The cell density in each well was subsequently determined by taking the median voxel cell number per mL.

|  | 6-well plates | 3×3 cubic-well plate | p-value |
| --- | --- | --- | --- |
| Cell density (10^6^ cells/mL) | 0.04 [0-0.16] | 0.12 [0.03-1.07] | <0.0001^a^ |

**Table S4** Well cell density (10^6^ cells/mL) in wells for 6-well plates and the 3×3 cubic-well plate. Data are presented as median [range]. ^a^ Mann-Whitney *U* test

13. **Detection probability calculations**

Cell numbers are indicated for a detection probability of 50 % and 90 % at background levels corresponding to 20, 40, 60, 80% of the radioactivity concentration in wells (6-well plate) with ^89^Zr-labeled cells

*6-well plate experiment*

| **Background levels** | **Cell numbers**  **(detection probability: 50 %)** | | **Cell numbers**  **(detection probability: 90 %)** | |
| --- | --- | --- | --- | --- |
| **6-well plates** | **PET/CT** | **PET/MRI** | **PET/CT** | **PET/MRI** |
| **20%** | 1.64×10^4^ | 1.64×10^4^ | 4.84×10^4^ | 8.79×10^4^ |
| **40%** | 8.36×10^4^ | 9.41×10^4^ | 1.91×10^5^ | 2.12×10^5^ |
| **60%** | 3.22×10^5^ | 3.70×10^5^ | 7.89×10^5^ | 7.66×10^5^ |
| **80%** | 1.28×10^6^ | 1.39×10^6^ | 2.31×10^6^ | 2.47×10^6^ |

**Table S5 Detection probability calculations for the 6-well plate experiment**

**References**

[1]	Carney JPJ, Townsend DW, Rappoport V, Bendriem B. Method for transforming CT images for attenuation correction in PET/CT imaging. Med Phys. 2006;33:976–83.
